# Reprogrammed lipid metabolism in advanced resistant cancers: an upcoming therapeutic opportunity

**DOI:** 10.20517/cdr.2024.131

**Published:** 2024-11-19

**Authors:** Mario Cioce, Mariamena Arbitrio, Nicoletta Polerà, Emanuela Altomare, Antonia Rizzuto, Carmela De Marco, Vito Michele Fazio, Giuseppe Viglietto, Maria Lucibello

**Affiliations:** ^1^Department of Medicine, Laboratory of Molecular Medicine and Biotechnology, University Campus Bio-Medico of Rome, Rome 00128, Italy.; ^2^Institute of Translational Pharmacology, National Research Council of Italy (CNR), Rome 00133, Italy.; ^3^Department of Biomedical Sciences, Institute for Biomedical Research and Innovation, National Research Council of Italy (CNR), Catanzaro 88100, Italy.; ^4^Department of Health Sciences, “Magna Graecia” University of Catanzaro, Catanzaro 88100, Italy.; ^5^Department of Medical and Surgical Sciences, “Magna Graecia” University of Catanzaro, Catanzaro 88100, Italy.; ^6^Department of Experimental and Clinical Medicine, “Magna Graecia” University of Catanzaro, Catanzaro 88100, Italy.

**Keywords:** Metabolic signaling, therapy resistance, tumor microenvironment, metastasis, immune evasion

## Abstract

Resistance of cancer to therapy is the main challenge to its therapeutic management and is still an unsolved problem. Rearranged lipid metabolism is a strategy adopted by cancer cells to counteract adversity during their evolution toward aggressiveness and immune evasion. This relies on several mechanisms, ranging from altered metabolic pathways within cancer cells to evolved dynamic crosstalk between cancer cells and the tumor microenvironment (TME), with some cell populations at the forefront of metabolic reprogramming, thereby contributing to the resistance of the whole ecosystem during therapy. Unraveling these mechanisms may contribute to the development of more effective combinatorial therapy in resistant patients. This review highlights the alterations in lipid metabolism that contribute to cancer progression, with a focus on the potential clinical relevance of such findings for the management of therapy resistance.

## INTRODUCTION

Intra-tumor heterogeneity is an established propelling factor of treatment resistance and, therefore, a critical determinant of tumor aggressiveness and therapy resistance^[1]^. Intra-tumor heterogeneity occurs at all levels: genomic, proteomic, lipidomic, and ultimately, metabolic. On the other hand, the tumor microenvironment (TME) is characterized by fluctuating oxygen levels, low pH, reduced metabolite availability, and accumulation of tumor-derived metabolites (metabolic waste). This contributes to the emergence of subpopulations of cells with different genotypes and phenotypes, forming a highly heterogeneous and dynamic ecosystem*.*

The goal of any successful therapy is to attenuate disease persistence and progression by targeting specific cancer cell subpopulations endowed with the ability to survive in stressful conditions^[2]^. Thus, characterizing the metabolic events that dynamically occur in a specific tumor and their TME counterpart can greatly aid in understanding and fighting the resistance to therapy.

In principle, cancer cells must be endowed with metabolic plasticity to process substrates differently. While this latter enables cancer cells to cope with adverse conditions, it also makes the cells dependent on specific metabolic pathways. Thus, metabolic reprogramming may indeed represent a vulnerability of cancer cells that can be exploited therapeutically. However, not all cancers share the same metabolic abnormalities, as variations between tumors lead to inter-tumor heterogeneity, which can compromise the efficacy of therapeutic approaches. For example, fatty acid synthase (FASN), a key enzyme in the lipogenesis pathway, is associated with varying outcomes in different cancer types. Elevated levels of FASN have been correlated with worse prognosis in liver and colorectal cancers^[3]^. Conversely, inhibiting FASN in lung cancer cells has been shown to promote tumor metastasis *in vivo*^[4]^. In addition, the metabolic state of a tumor is influenced by the general condition of the patient (e.g., age, gender, comorbidities, iatrogenic conditions).

Another important point to consider is the tissue-specific metabolic profile, which prevents the establishment of a general metabolic scenario for tumors arising in different parts of the body or for primary tumors targeting different organs (e.g., metastatic ones).

This suggests the importance of developing therapeutic approaches targeting tumor-specific metabolic pathways, taking into account their different molecular phenotypes.

Recent evidence indicated that reprogramming lipid metabolism is a strategy adopted by cancer cells to survive under stressful conditions and to counteract adversity during their evolution toward stages of increased cellular aggressiveness, therapy resistance, and immune evasion.

Lipid metabolism is essential not only for producing membrane biomass but also for modulating the functional properties of cell membranes. In particular, the lipid composition of the cell membrane in cancer cells differs significantly from that of normal cells and promotes aberrant activation of oncogenic signaling pathways involved in tumor progression and drug resistance. In this review, we discuss how alterations in lipid metabolism and aberrant signaling pathways are closely linked. For instance, elevated cholesterol levels in lipid raft domains, which are specialized centers for the assembly of biomolecules, induce aberrant phosphoinositide 3-kinase (PI3K)/AKT signaling activation, thereby promoting chemoresistance and tumor progression^[5]^. By modulating the structural properties of cell membranes, lipids may promote an epithelial-to-mesenchymal transition (EMT) phenotype correlated with resistance to therapy^[6]^, highlighting lipids as crucial drivers of tumor malignancy.

We also discuss how metabolic reprogramming toward lipid metabolism, including de novo lipogenesis, fatty acid (FA) oxidation, and lipid uptake, is associated with tumor aggressiveness. For example, alteration of cholesterol biosynthesis is a key event in cancers at high risk of early progression but is also a potential target in chemoresistant cancers. Furthermore, after outlining how the reprogramming of lipid metabolism represents a key metabolic adaptability of therapy-resistant cancer cells, we discuss how this results in a dysfunctional immune phenotype. This results in immune evasion and resistance to immunotherapy.

Finally, we discuss that alterations in lipid metabolism in cancer cells are exacerbated in the context of obesity, highlighting that the crosstalk between cancer cells and surrounding cells in the TME is driven by the activation of different molecular circuits and mechanisms, which in turn dictate the progression of cancer toward an increasingly aggressive and immune-evasive phenotype. This is relevant when growth and metastasis occur predominantly in a lipid-rich environment.

## MEMBRANE LIPID REMODELING AND THERAPY RESISTANCE

In addition to being an important source of energy, lipids are a large and complex group of biomolecules that, by altering the properties of cell membranes, influence oncogenic signaling pathways involved in tumor progression and drug resistance.

Cell membrane properties are influenced by a variety of factors, including lipid composition, FA chain length, degree of unsaturation, and cholesterol concentration. Interactions between sphingolipids, saturated lipids, and cholesterol are favored over interactions with highly unsaturated lipids, resulting in the formation of tightly packed, ordered domains, called lipid raft domains, which are dispersed throughout the plasma membrane. Conversely, polyunsaturated or very short lipids form disordered and irregularly packed domains, contributing to the stabilization of phase separation^[7]^. Lipid raft domains are centers for the assembly of signaling molecules involved in the transduction of signaling pathways critical for cancer cell growth and development. These include the insulin-like growth factor (IGF) system, the Ras/ERK/mitogen-activated protein kinase (MAPK) pathway, the PI3K/AKT pathway, and the TGF-beta signaling pathway, which are frequently deregulated in cancer cells and associated with resistance to therapy^[8,9]^, suggesting that lipid metabolism and signaling pathways are closely linked.

**Figure 1:**
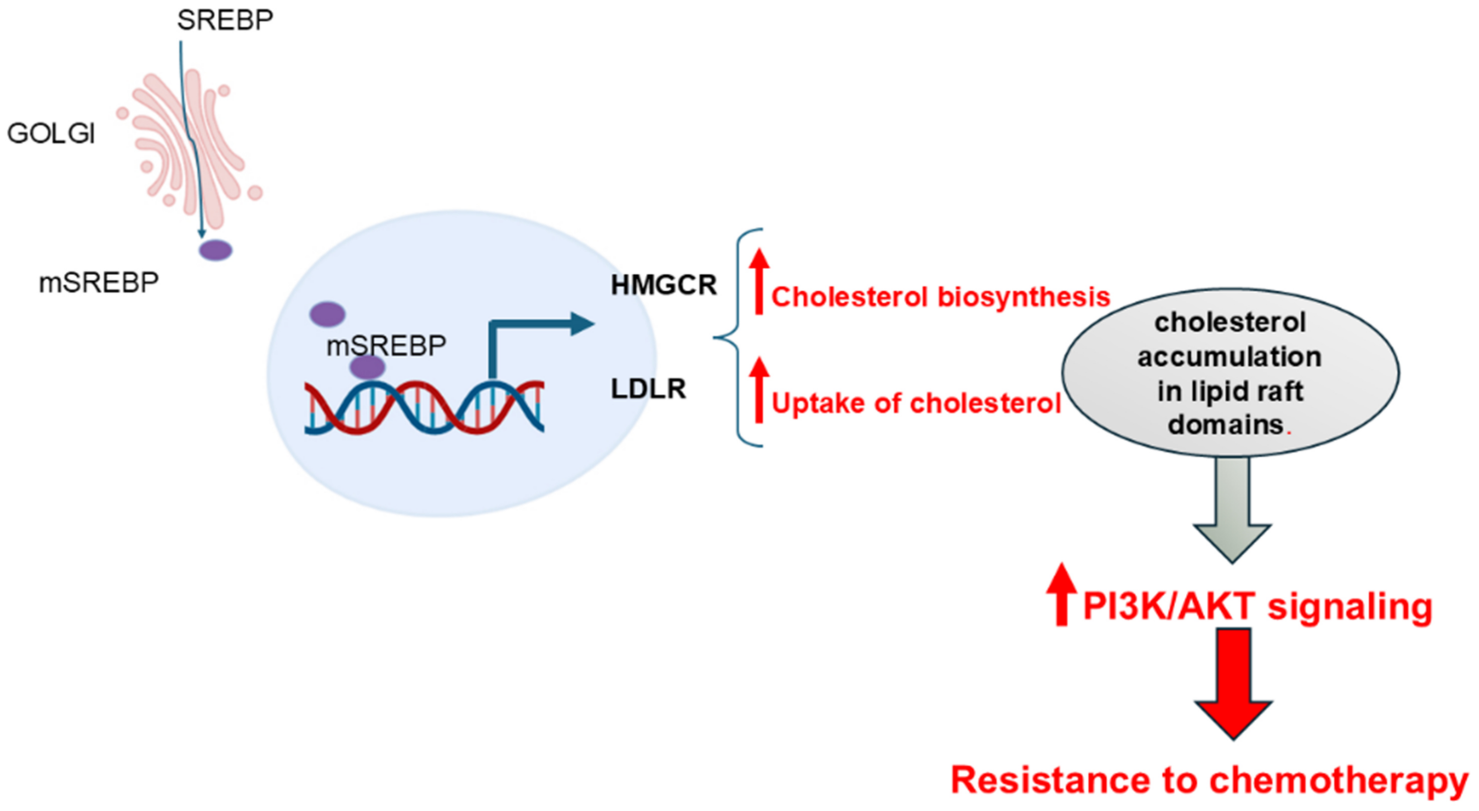
Cholesterol metabolism promotes chemoresistance. The SREBPs are a family of transcription factors. The mature active form of SREBP is generated within the Golgi apparatus by the cleavage of the precursor protein. Active forms of SREBP then translocate to the nucleus, where they promote transcription of genes involved in FA synthesis and cholesterol metabolism, including HMGCR and LDLR. HMGCR stimulates the synthesis of cholesterol. LDLR facilitates the internalization of cholesterol-carrying lipoproteins into cells. This results in the accumulation of cholesterol, predominantly within lipid raft domains, which, in turn, promotes AKT signaling and resistance to chemotherapy. Created with BioRender.com. HMGCR: 3-Hydroxy-3-methylglutaryl coenzyme A reductase; LDLR: low-density lipoprotein receptor; PI3K: phosphoinositide 3-kinase; AKT: protein kinase B.

Cholesterol is a key component of lipid raft domains. Cholesterol metabolism has been shown to be involved in the regulation of PI3K/Akt signaling, thereby influencing resistance to therapy-induced cell death in cancer cells^[5]^. Yu *et al.* have shown that cellular retinoic acid binding protein II (CRABP II) induced sterol-regulatory-element-binding-proteins-1c (SREBP-1c) in pancreatic ductal adenocarcinoma (PDAC) cells. SREBPs are a family of transcription factors implicated in FA and cholesterol biosynthesis. Specifically, SREBP-1c promoted the expression of 3-hydroxy-3-methylglutaryl coenzyme A reductase (HMGCR), the rate-limiting enzyme in cholesterol biosynthesis, and low-density lipoprotein receptor (LDLR), which is responsible for cellular uptake of cholesterol-carrying lipoproteins. Aberrant expression of CRABP II induced cholesterol accumulation primarily in lipid raft domains, thereby promoting AKT activation and resistance to gemcitabine [Figure 1]. Consistently, CRABP II was highly expressed in samples of PDAC and associated with poor survival^[10]^. Consistent with these findings, statin treatment was correlated with a reduced risk of all-cause mortality in PDAC patients^[11]^ [Table 1].

**Table 1:** The role of cell membrane lipids in aggressive tumors

**Change in membrane lipid composition**	**Effect on cancer cells**	**Ref.**
↑ Cholesterol	↑ PI3K/Akt signaling ↑ Resistance to chemotherapy ↓ Prognosis in cancer patients	[10-12]
↑ Phospholipid	↑ EGFR signaling ↑ Resistance to chemotherapy ↓ prognosis in cancer patients	[13-15]
↑ GPs	↑ EMT ↑ Metastasis	[21]
↑ A-series gangliosides	↓ Inhibited TGF-beta signaling ↓ EMT ↑ Cancer patient survival ↑ CD8 T cell dysfunction	[23,24]

PI3K: Phosphoinositide 3-kinase; EGFR: epidermal growth factor receptor; GPs: glycerophospholipids; EMT: epithelial-to-mesenchymal transition.

**Figure 2:**
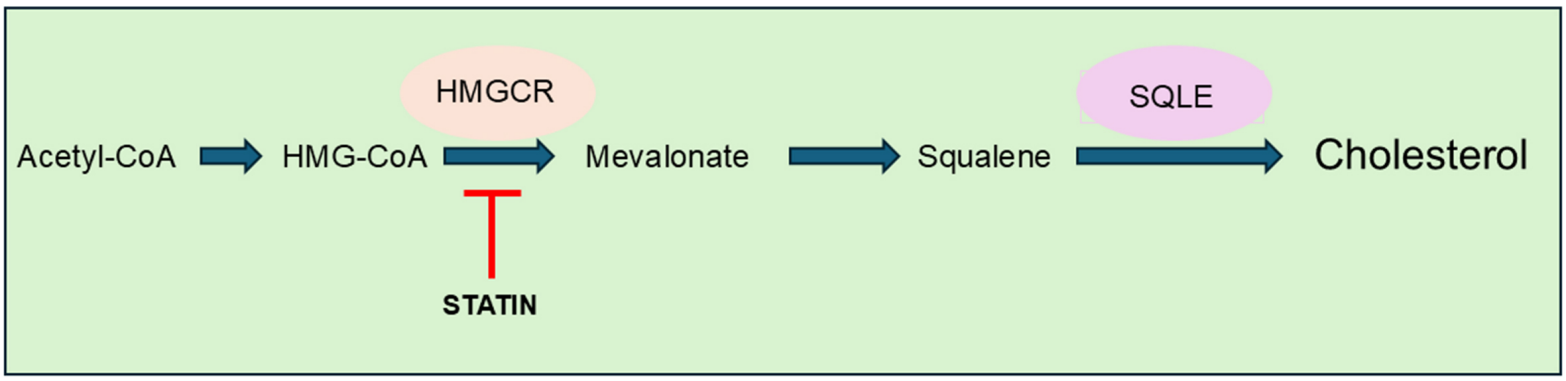
A simplified representation of the cholesterol biosynthesis pathway. A simplified representation of the cholesterol biosynthesis pathway. The process begins with acetyl-CoA. Subsequent reactions involve the union of acetyl-CoA and acetoacetyl-CoA, resulting in the formation of HMG-CoA. HMGCR then facilitates the reduction of HMG-CoA, resulting in the production of mevalonate and then squalene, which ultimately leads to the production of cholesterol. HMGCR is the rate-limiting enzyme in the cholesterol biosynthesis pathway. SQLE plays a critical role in the mevalonate-cholesterol pathway. Statins inhibit HMGCR, preventing the synthesis of mevalonate and cholesterol. HMGCR: 3-Hydroxy-3-methylglutaryl coenzyme A reductase; SQLE: squalene epoxidase.

Further, by enhancing de novo cholesterol biosynthesis [Figure 2], squalene epoxidase (SQLE) promoted the activation of downstream PI3K/Akt and induced cisplatin resistance in head and neck squamous cell carcinoma (HNSCC)^[12]^ [Table 1].

Growth factor signaling can also affect plasma membrane properties. An elegant work by Bi *et al.* showed that constitutive activation of epidermal growth factor receptor (EGFR) upregulated lysophosphatidylcholine acyltransferase 1 (LPCAT1) and promoted continuous oncogene activation cancer cells^[13]^ [Table 1]. LPCAT1 plays a role in remodeling plasma membrane structure by altering phospholipid composition. Elevated levels of LPCAT1 were associated with poor outcomes in cancer patients and induced resistance to gefitinib in lung adenocarcinoma cells (LUAC)^[14]^ and to paclitaxel in breast cancer^[15]^.

Thus, cell membrane remodeling in tumor cells may have important functional implications in driving malignant progression and treatment resistance. The identification of pathways regulated by specific lipids may contribute to the development of effective targeted therapies in patients unresponsive to conventional chemotherapy.

### Cell membrane lipids and the EMT process: a mutual interaction toward therapy resistance

The acquisition of a reprogrammed metabolic state in cancer cells could be a primal change leading to EMT.

Using Raman spectroscopy, Sabtu *et al.* performed a metabolomic analysis of human breast cancer samples and showed that EMT-enriched tissues had a higher lipid content compared to their EMT-less counterparts, highlighting lipids as key players of biochemical changes in the EMT process^[16]^. This is relevant considering the close relationship between the acquisition of EMT or “EMT-hybrid” states and resistance to therapy observed in many tumor settings^[17,18]^.

EMT is a critical step in the metastatic process whereby cells acquire a motile and invasive phenotype characterized by loss of contact with neighboring cells, changes in cell shape and morphology, and alterations in the biophysical properties of the cell membrane.

EMT requires destabilization of lipid raft domains and an increase in plasma membrane fluidity^[7]^. Several observations have shown that cholesterol was essential for maintaining proper membrane fluidity and the EMT-associated drug resistance phenotype in cancer cells^[19]^, consistent with data showing that inhibitors of cholesterol synthesis disrupted the lipid raft domain and sensitized lung cancer cells to paclitaxel^[19]^.

Estrogen-related receptor alpha (ERRα) has been identified as a key player in the development of obesity-related tumors, including breast cancer^[20]^. It can be targeted by the transcription factor EB (TFEB), which has been proposed as a novel target for lipid metabolism disorders. The TFEB-ERRα axis induced an increase in glycerophospholipids (GPs) containing unsaturated fatty acids (UFAs). This resulted in increased cell membrane fluidity, thereby promoting the EMT program in endometrial cancer (EC) cells [Table 1]. This is consistent with the observation that both TFEB and ERRα levels were positively correlated with dyslipidemia and metastasis in EC patients^[21]^. More generally, the latter evidence links PUFA dynamics to cancer resistance and progression. Interestingly, ERRα promoted the survival of epidermal growth factor receptor 2 (HER2)-positive breast cancer cells resistant to lapatinib, suggesting that ERRα inhibition may be an effective adjuvant therapy in HER2-positive breast cancer with poor outcome^[20]^. TFEB has been shown to mediate resistance to mTOR inhibition in renal cell carcinoma (RCC) and to increase programmed death ligand-1 (PD-L1) expression in cancer cells, thereby promoting immune evasion^[22]^.

Other key molecules regulating TGF-β-induced EMT processes are sphingolipids. Zhang *et al.* have shown that ST3-beta-galactoside alpha-2,3-sialyltransferase 5 (ST3GAL5) is a key enzyme responsible for the synthesis of a-series gangliosides, which are the major sphingolipids in lipid raft domains^[23]^. By promoting the localization and degradation of the TGF-β type I receptor, they played a regulatory role during the EMT process in lung cancer cells. Consistently, low levels of ST3GAL5 correlated with poor prognosis in lung and bladder cancer patients^[23]^. However, in clear cell renal cell carcinoma (ccRCC), high expression of ST3GAL5 correlated with a dysfunctional state of CD8^+^ cells and predicted poor clinical outcome^[24]^, and in avian neural crest cells, sphingolipids metabolism promoted the activation of the Wnt and bone morphogenetic protein (BMP) pathways, which are critical for the development of the EMT process, suggesting that the role of sphingolipids may differ depending on the tumor type^[25]^.

In conclusion, these findings suggest that reprogramming of lipid metabolism by altering the structural and functional properties of cell membranes may promote an EMT-associated drug resistance phenotype in cancer cells. The identification of potential key players may open the way to molecularly targeted therapy and provide valuable insights into how metabolic vulnerability influences chemoresistance, as well as preclinical evidence that targeting lipids may improve response to chemotherapy.

## CHOLESTEROL METABOLISM IN AGGRESSIVE CANCER CELLS

In addition to its role in determining cell membrane properties, cholesterol metabolism may be a critical biomarker for detecting highly aggressive cancer cells.

Recently, a preclinical study by Muta *et al.* highlighted the importance of cholesterol metabolism in colorectal precancerous lesions called serrated polyps, which contain large numbers of metaplastic cells with invasive and therapy-resistant behavior^[26]^. Using publicly available human RNAseq data, the authors found that human CRCs showed higher enrichment in cholesterol signatures than conventional CRCs. Furthermore, the increased activity of SREBP2 induced an increase in cholesterol metabolism [Figure 1] and led to the development of aggressive tumors resistant to standard therapy.

Consistently, inhibition of cholesterol metabolism prevented the progression of refractory malignant cells. This suggests that alterations in cholesterol metabolism are key events driving tumor progression and points to SREBP2 as a potential selective, actionable target in aggressive serrated cancer cells. SREBP2 has also emerged as a clinically relevant biomarker to improve the stratification of disease subsets and to specifically detect highly aggressive preneoplastic serrated lesions that are refractory to conventional therapy^[26]^.

In line with this, a fascinating study by Chen *et al.* showed that mice with deletions in both the PTEN and PML tumor suppressor genes developed metastatic prostate disease characterized by upregulation of SREBPs. Notably, fatostatin, a specific inhibitor of SREBPs, reduced the transcription of lipogenic genes, and inhibited tumor growth and distant lymph node metastasis. Consistent with these data, a significant enrichment of the SREBP-1 signature was identified in metastatic samples compared to primary samples^[27]^. SREBP-mediated cholesterol and FA synthesis has also been shown to be upregulated in glioma cells resistant to temozolomide (TMZ). TMZ is used as a standard therapy for glioblastoma multiforme (GBM). Interestingly, alteration of lipid metabolism led to a change in cell shape in resistant cells^[28]^, suggesting that response to therapy can be predicted by analyzing cancer cell morphology, as previously shown in cancer cells resistant to inhibitors of the ErbB receptor tyrosine kinase (RTK) family^[29]^.

Taken together, these findings implicate SREBP-mediated cholesterol as a key player in tumor progression to metastatic disease. Alterations in cholesterol metabolism could be detected in highly aggressive cells before neoplastic transformation, highlighting the importance of cholesterol metabolism as a potential biomarker of tumors at high risk of progression early on. Aberrant cholesterol biosynthesis increases cholesterol production and renders cancer cells dependent on this pathway, providing a therapeutic opportunity in cancer cells refractory to conventional therapy.

## DE NOVO LIPOGENESIS AND STORAGE IN THERAPY-RESISTANT TUMORS

**Figure 3:**
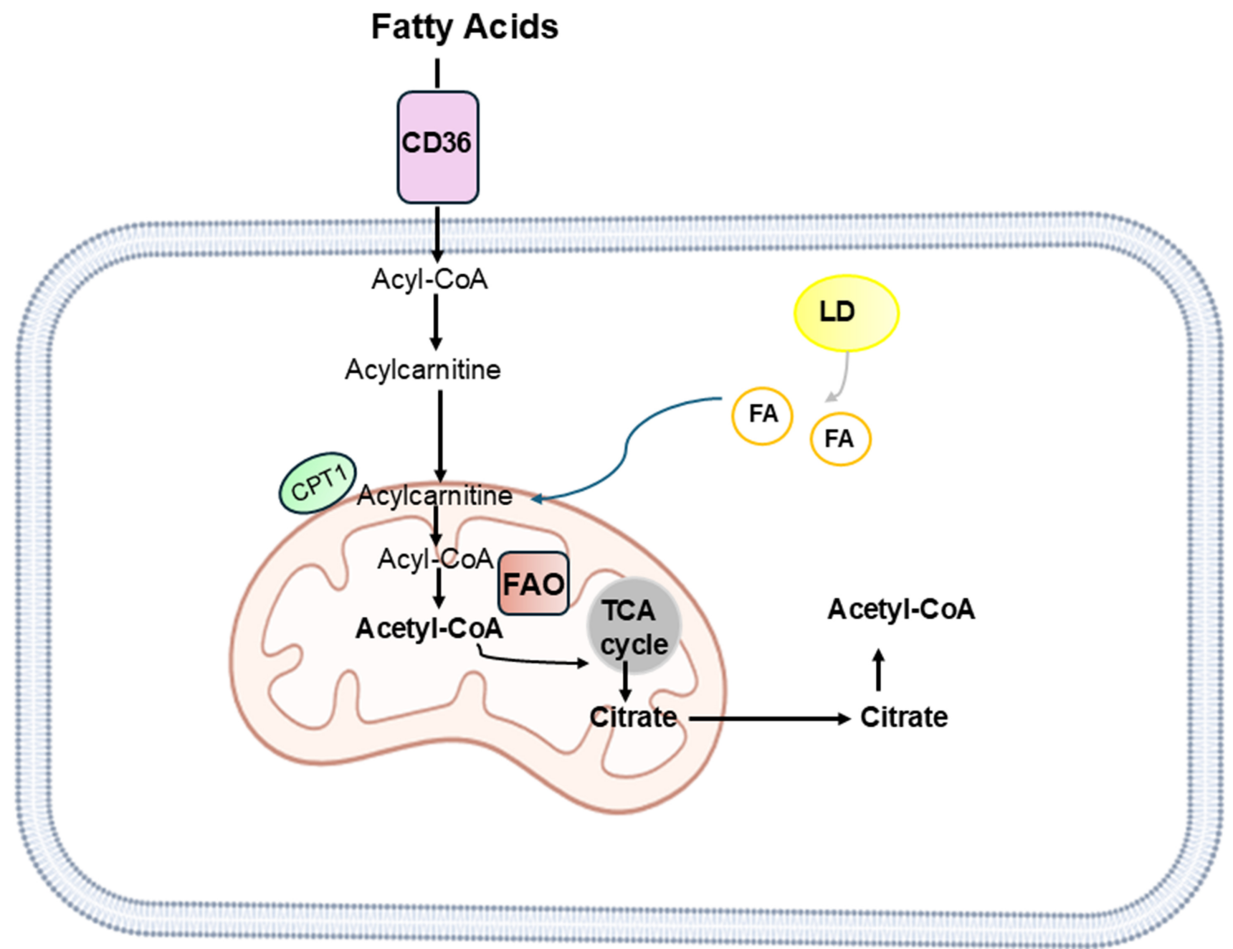
A simplified representation of FAO. Mitochondrial FAO is a catabolic process that breaks down long-chain FAs to produce acetyl-CoA. The long-chain FA requires the carnitine shuttle system to deliver long-chain FA from the cytoplasm to the mitochondria. CPT1 in the outer mitochondrial membrane controls the entry of FAs into the mitochondria. FA-CoA is metabolized to an acylcarnitine derivative; acylcarnitine is then reconverted to FA-CoA in the mitochondria, where it undergoes a series of dehydrogenation reactions to form acetyl-CoA. CoA then enters the TCA cycle to make ATP. LDs release FAs to form acyl-CoA. Created with BioRender.com. FAO: β-Fatty acid oxidation; FAs: fatty acids; CPT1: carnitine palmitoyltransferase 1; TCA: tricarboxylic acid cycle; ATP: adenosine triphosphate; LDs: lipid droplets.

Increased lipid accumulation induces the formation of storage organelles called lipid droplets (LDs), which play an important role in balancing de novo lipogenesis and β-fatty acid oxidation (FAO). By releasing lipids for energy production during starvation, LDs are an important resource for the high energy demands of cancer cells during the metastatic process. They also play a critical role in mediating reactive oxygen species (ROS) production. The release of lipids fuels the FAO pathway, a major source of ATP that occurs primarily in mitochondria [Figure 3].

**Figure 4:**
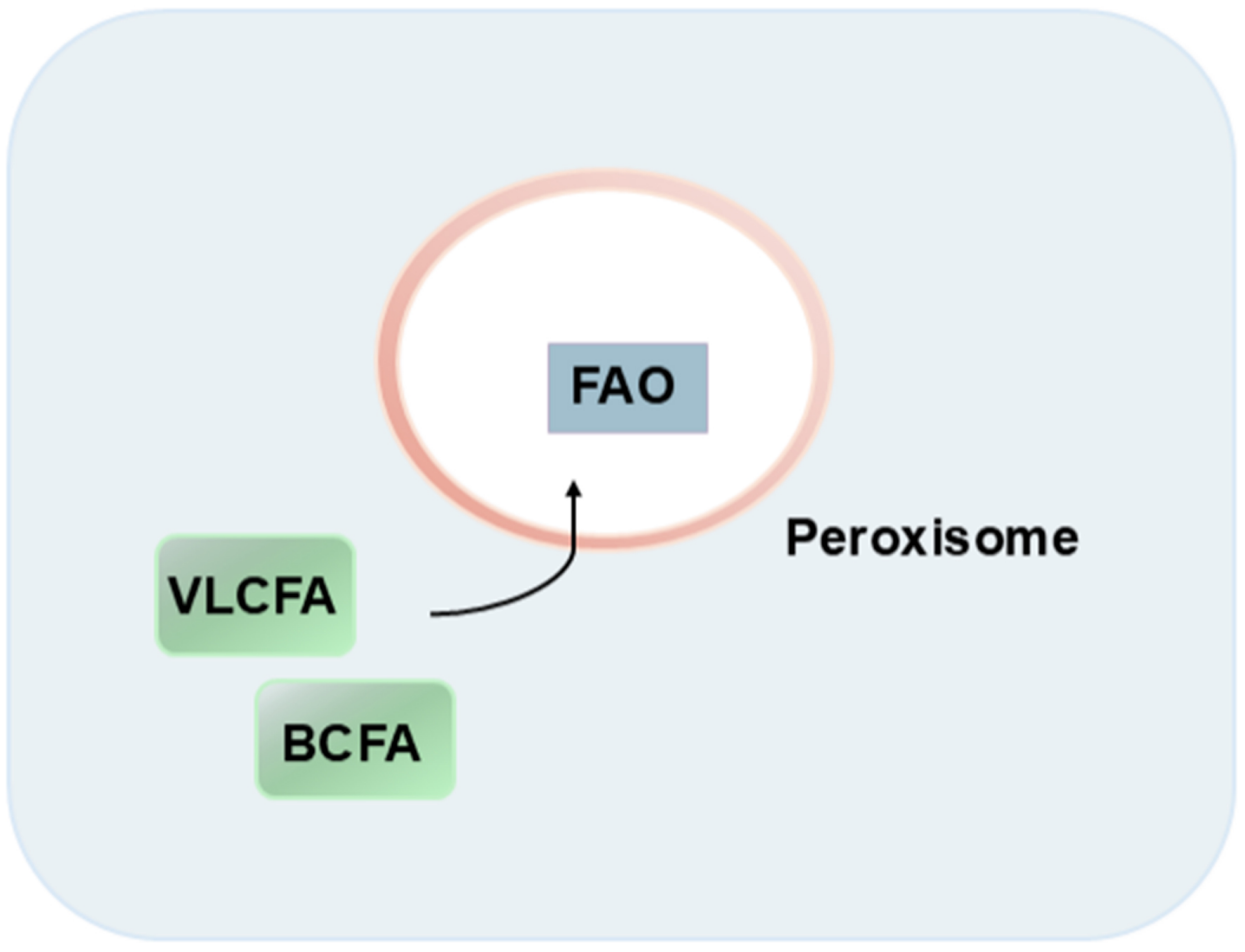
A simplified representation of Peroxisome. Peroxisomes are membrane-bound organelles. They contain enzymes such as catalase and peroxidase, which are needed to break down FAs and hydrogen peroxide. Complex lipids are broken down in peroxisomes. FAs: Fatty acids; VLCFA: very long chain fatty acid; FAO: β-fatty acid oxidation; BCFA: branched-chain fatty acid.

However, catabolism of complex lipids, such as very long chain fatty acids (VLCFAs) and branched-chain fatty acids (BCFAs), occurs in peroxisomes, which are also scavenger organelles involved in the conversion of ROS [Figure 4]. Thus, LDs and peroxisomes work together to control lipolysis and ROS^[30]^.

**Figure 5:**
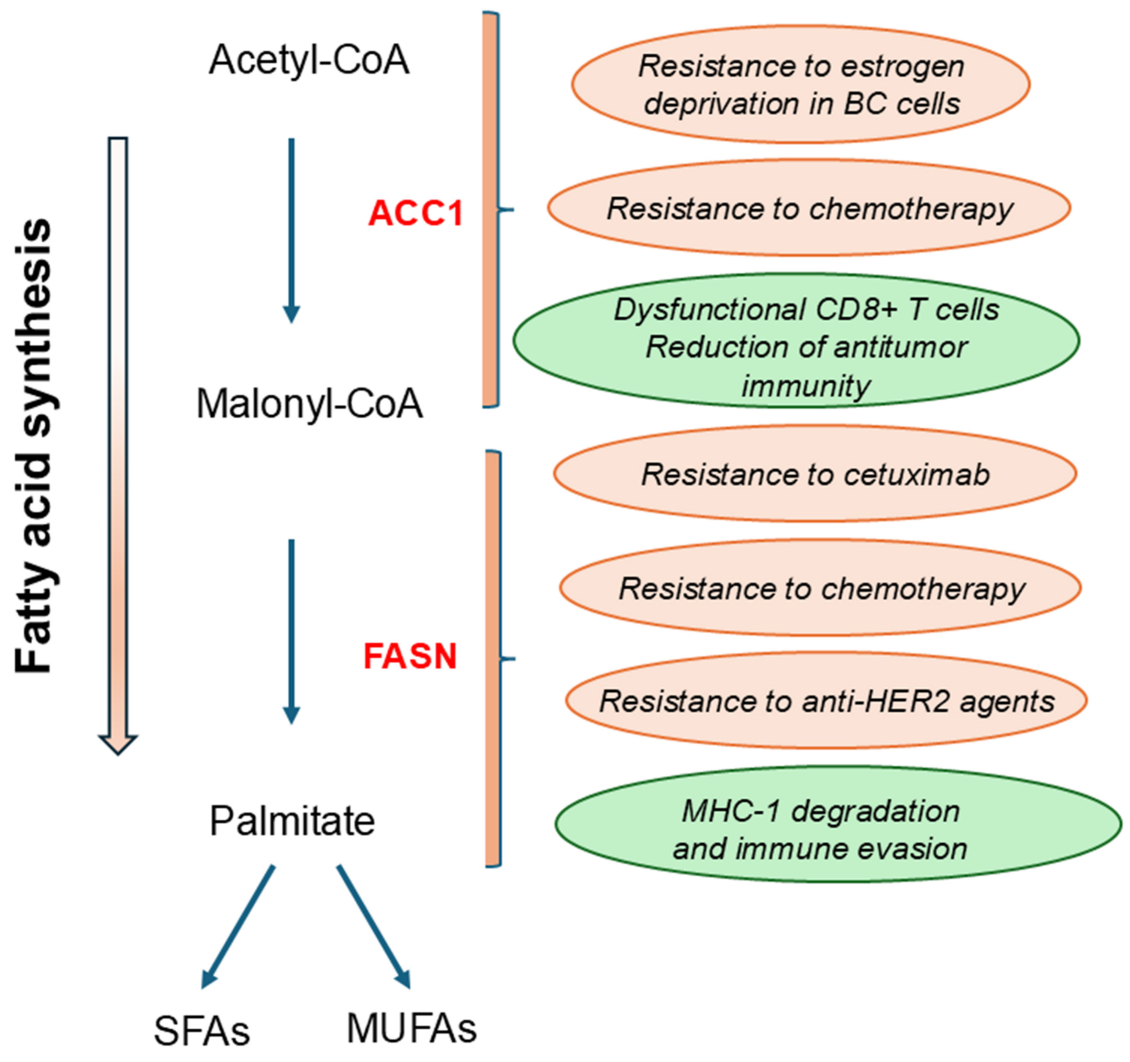
ACC1 and FASN as potential therapeutic targets for overcoming treatment resistance. A simplified representation of Fa synthesis provides phospholipids for the cell membrane. ACC1 is a critical enzyme in the carboxylation of acetyl-CoA to malonyl-CoA. Malonyl-CoA is then converted by FASN to palmitate, a saturated FA. Palmitate undergoes additional modifications, resulting in the production of SFA and MUFA. ACC1: Acetyl-CoA carboxylase 1; FASN: fatty acid synthase; FA: fatty acid; HER2: epidermal growth factor receptor 2; MHC-I: major histocompatibility complex class I.

Recently, increased intracellular lipid storage and peroxisomes have been shown to be critical events in controlling redox homeostasis and metabolic adaptability in tumors resistant to endocrine therapy. In this context, acetyl-CoA carboxylase 1 (ACC1) is a key metabolic player. ACC1 is a critical enzyme in the carboxylation of acetyl-CoA to malonyl-CoA, an important substrate for the formation of complex FAs [Figure 5]^[31]^.

Using breast cancer models of aromatase inhibitor resistance, Bacci *et al.* showed that nuclear factor kappa B (NF-κB) activation induced aberrant lipid metabolism characterized by increased intracellular lipid deposition, which was maintained by either novo FA synthesis or CD36-mediated VLCFA/BCFA uptake^[31]^. The release of lipids from LDs was utilized in the FOA process to support the energetic capacity of endocrine-resistant ER+ breast cancer cells. Metabolic reprogramming was achieved through ACC1, consistent with literature data suggesting that increased ACC1 abundance may be a common feature of endocrine-resistant models^[32]^ [Table 2]. Targeting ACC1 with 5-tetradecyloxy-2-furoic acid (TOFA, an inhibitor of ACC) reduced LD levels and induced a specific inhibitory effect on resistant breast cell growth through an increase in ROS and lipid peroxidation. The addition of exogenous complex lipids or ROS scavengers reversed this inhibitory effect, suggesting a functional role for peroxisomes in metabolic plasticity. Furthermore, patients responding to the aromatase inhibitor letrozole showed decreased peroxisome-related gene signatures after 90 days of treatment^[31]^. This suggests that peroxisomal activities may be involved in acquired rather than de novo resistance.

**Table 2:** Lipids fuel aggressive cancer cells

**Changes**	**Effects**	**Ref.**
↑ ACC1	↑ Resistance to endocrine therapy ↑ Cetuximab resistance ↓ CD8^+^ T cell cytotoxicity ↑ Immune evasion	[31,33,35]
↑ FASN	↑ Resistance to chemotherapy ↑ Resistance to anti-HER2 agents ↓ CD8^+^ T cell cytotoxicity ↑ Treg function ↓ Efficacy of anti-PD-L1 therapy	[40-43,45]
↑ CD36	↑ Tumor progression ↑ Metastasis ↓ Prognosis in cancer ↑ Resistance to chemotherapy ↑ Resistance to anti-PD-1 treatment ↑ Treg function	[51,53-57,59,61,63]

ACC1: Acetyl-CoA carboxylase 1; HER2: epidermal growth factor receptor 2; FASN: fatty acid synthase; PD-L1: programmed death ligand-1; PD-1: programmed cell death protein 1.

Interestingly, the level of total ACC, predominantly ACC1, was a critical factor in the reprogramming of cancer metabolism in HNSCC cells with acquired cetuximab resistance, shifting the dependence from glycolysis to lipogenesis. These findings were further validated by an analysis of tumor samples from cetuximab-treated HNSCC patients. Notably, the combination of TOFA with cetuximab resulted in significant inhibition of cell growth in cetuximab-resistant HNSCC *in vivo* models^[33]^ [Table 2].

In addition, ACC1 activities and LD assembly have been shown to be critical events in mediating resistance to chemotherapy in esophageal cancer cells^[34]^.

While ACC1 supported cancer cell growth, its activity has been shown to impair T cell activities, as recently demonstrated in an elegant study by Hunt *et al.*^[35]^.

The dysfunctional state in CD8^+^ cells was characterized by forced ACC1 activities, which in turn led to cytosolic FA synthesis and the formation of high levels of LDs due to the inability to utilize or degrade saturated lipids such as palmitic and stearic acids, which form solid lipid deposits. The high activity of ACC1 was induced by nutrient deprivation or hypoxic stress and resulted in a dramatic inhibition of FAO. FAO is a critical energetic support of T cells that enhances antitumor activities under nutrient deprivation, as demonstrated in many preclinical and clinical studies. Consistent with these observations, inhibition of ACC1 activities resulted in the generation of T cells capable of effective antitumor activity^[35]^.

Taken together, these findings suggest that reprogramming of lipid metabolism is a critical metabolic adaptability in resistant cancer cells. On the other hand, alterations in lipid metabolism in immune cells often lead to a dysfunctional phenotype, thereby promoting progression. This suggests the importance of investigating a broader panel of cellular targets for successful therapy and exploring the complex landscape of the TME and its potential for combined targeted therapy.

### FASN and drug resistance

Increased de novo synthesis of FAs is altered in cancer cells and is associated with chemoresistance^[36,37]^. FASN is a key enzyme in this process [Figure 5]. Its expression is modulated by the transcription factor SREBP1, particularly through dysregulation of signaling pathways critical for tumor progression, such as PI3K/AKT^[37]^. In addition, KRAS mutations have been shown to increase FASN expression in LUAC, which in turn promoted the synthesis of monounsaturated fatty acids (MUFAs) and saturated fatty acids (SFAs) [Figure 5], thereby preventing ferroptosis in cancer cells^[38]^, a form of cell death induced by the peroxidation of polyunsaturated membrane phospholipids^[39]^. FASN upregulation induced resistance to cisplatin in breast and ovarian cancer (OvCA) cells. Consistently, FASN inhibition increased the chemosensitivity of cancer cells to cisplatin^[40,41]^ [Table 2].

Interestingly, FASN upregulation correlated with oxaliplatin resistance and poor prognosis in CRC patients. Consistent with these data, upregulation of the MAPK/ERK and PI3K/AKT pathways by FASN-induced oxaliplatin resistance in CRC cells [Table 2]. In contrast, inhibition of FASN by orlistat or C75 enhanced the effect of oxaliplatin in cancer cells^[42]^. In GC cells, a positive feedback loop between HER2 and FASN resulted in resistance to the anti-HER2 agent trastuzumab, which was reversed by the combination of HER2 and FASN inhibitors. Interestingly, overexpression of FASN correlated significantly with a stemness gene signature and poor prognosis in patients with HER2+ GC, highlighting its role in cancer malignancy^[43]^ [Table 2].

Several observations have shown that upregulation of FASN activity dramatically impaired antitumor response^[44]^. Major histocompatibility complex class I (MHC-I) plays a critical role in the induction of an effective antitumor immune response by presenting antigens to T cells. A recent study by Huang *et al.* showed that MHC-1 is palmitoylated by FASN and degraded in the lysosomal compartment^[45]^. Interestingly, the combination of two different FASN inhibitors with an anti-PD-L1 antibody enhanced the efficacy of immunotherapy and induced a dramatic inhibition of tumor growth *in vivo*. Consistent with these data, lower FASN levels correlated with higher percentages of cytotoxic CD8^+^ T cells in hepatocellular carcinoma (HCC) patients^[45]^ [Table 2]. In contrast, FASN-mediated upregulation of lipid synthesis promoted the functional maturation of Treg cells, thereby suppressing the antitumor immune response and promoting colon cancer growth^[46]^.

In summary, several observations indicate that FASN upregulation is a critical event in malignant progression and the development of therapeutic resistance. In addition, FASN upregulation can induce severe immune cell dysfunction and promote an immune-evasive environment. Therefore, targeting FASN may enhance antitumor immunity and represents a promising approach for tumor immunotherapy. Currently, the oral FASN inhibitor TVB-2640 has shown promising results in clinical trials in advanced tumors^[47]^.

## CD36, FA UPTAKE AND CANCER RESISTANCE

Several factors influence the balance between lipid synthesis and uptake in cancer cells. For example, glucose deprivation^[48]^ or TME acidity^[49]^ can trigger β-oxidation of FAs to produce ATP, whereas a lipid-enriched environment can promote lipid uptake and storage^[30,50]^.

Extracellular free FAs are internalized by specialized transporters on cancer cells, and their expression is regulated by complex signaling pathways. Among these, CD36 is a well-characterized transmembrane glycoprotein belonging to the class B scavenger receptor family. Emerging clinical evidence has shown that high levels of CD36 or its associated signature are correlated with malignancy and poor prognosis in several cancers^[51]^ [Table 2].

**Figure 6:**
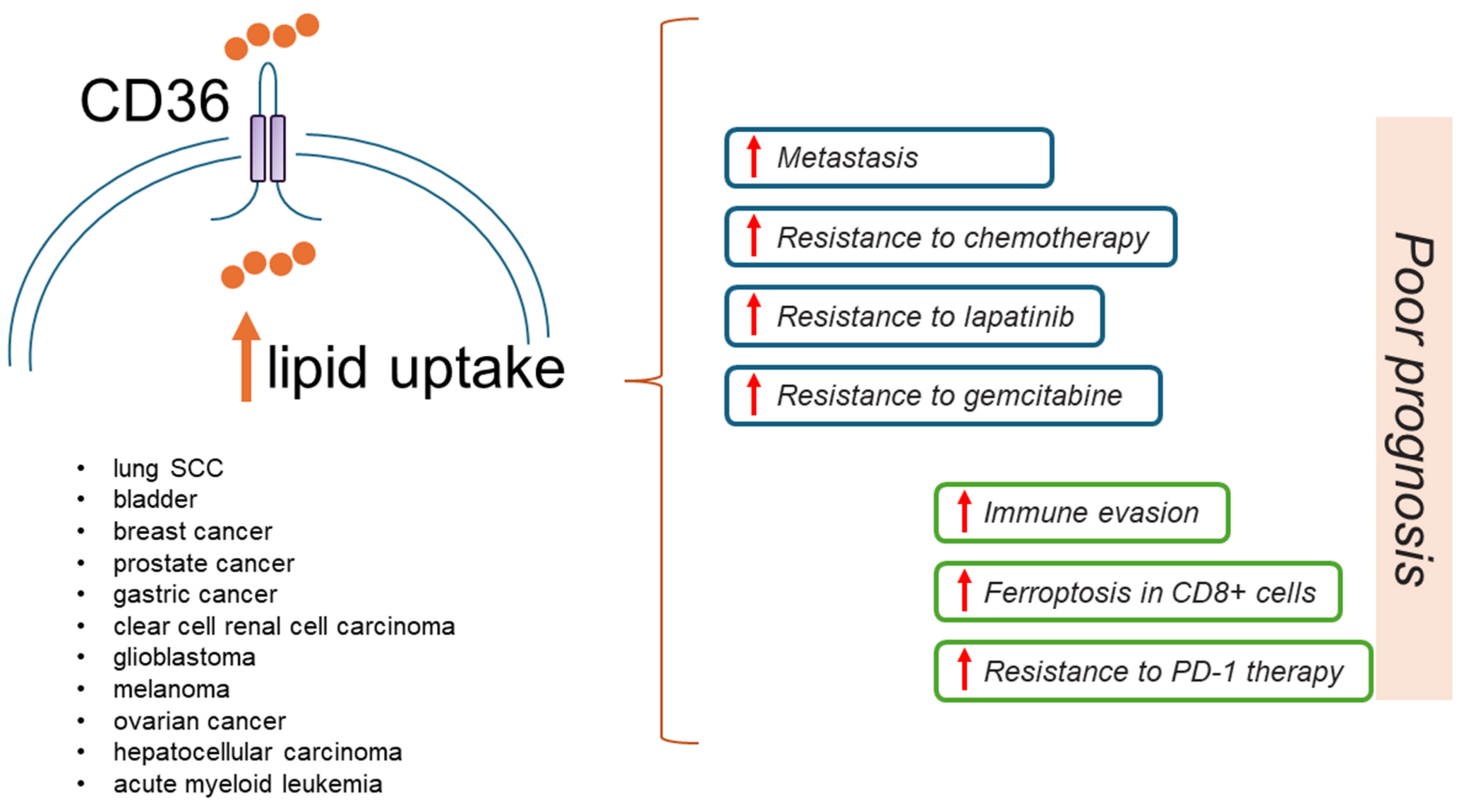
CD36 is a crucial target in aggressive and resistant tumors. A simplified representation showing that high levels of CD36 are correlated with malignancy, poor prognosis, and resistance to therapy in several cancers. CD36 is a FA transporter. FA: Fatty acid; SCC: squamous cell carcinoma; PD-1: programmed cell death protein 1.

Consistent with these findings, a recent pan-cancer analysis of enzymes, signaling, or transcription factors involved in glucose or FA metabolism showed that CD36 expression was associated with an EMT phenotype and was upregulated in several metastatic tumors [Figure 6].

In contrast, no significant difference was observed in the levels of glycolysis-related genes between primary and metastatic tumors^[52]^.

In prostate cancer cells, CD36 levels are unregulated in metastatic tissues compared to matched primary tumors, and specifically, its inhibition reduced the number of metastases and tumor growth *in vivo* xenograft models of prostate metastasis, consistent with findings that dietary lipids may promote metastasis^[53]^. Upregulation of CD36 in OvCA cells caused a metabolic shift toward dependence on exogenous FAs and cholesterol, which was mediated by the downregulation of both factors involved in FA synthesis, such as ACACA and SREBPs. Consistent with these findings, CD36 was a key driver in promoting OvCA progression, as demonstrated in an *in vivo* xenograft metastatic model of OvCA^[54]^ [Table 2].

Moreover, a high-fat diet promoted peritoneal tumor growth in a mouse model of GC transplanted with CD36-overexpressing cells. Consistently, high levels of CD36 correlated with poor prognosis in GC patients with peritoneal metastases^[55]^ Table 2, indicating that FA uptake is a critical event driving metastasis and correlated with poorer prognosis.

Consistent with these findings, cisplatin-resistant OC cancer cells increased the uptake of exogenous FAs and underwent lipid metabolic reprogramming from glycolysis to FAO, thereby promoting cancer survival under cisplatin-induced oxidative stress.

Specifically, oxidative stress induced by cisplatin treatment resulted in the depletion of intracellular antioxidants, such as NADPH. This, in turn, led to the inhibition of de novo lipogenesis, which requires NADPH. The reduction in de novo lipogenesis led to a decrease in malonyl-CoA levels, which acted as an allosteric inhibitor of carnitine palmitoyltransferase 1 (CPT1). CPT1 is a key enzyme involved in the transport of long-chain FAs into mitochondria [Figure 3]. Consequently, CPT1 activity promoted FAO. Consistent with these findings, CPT1 inhibition consistently resulted in impaired FAO and increased sensitivity of cisplatin-resistant OC cells to cisplatin treatment.

Interestingly, the increase in FAO activity was also observed in other tumor types following cisplatin treatment. This suggests that the combination of FAO inhibitors and platinum drugs may be an effective approach in cancer therapy to overcome chemotherapy resistance^[56]^.

Furthermore, breast cancer cells unresponsive to the HER2 inhibitor lapatinib exhibited high CD36 expression, which in turn induced metabolic rewiring by increasing FA uptake.

Reducing CD36 expression restored sensitivity to lapatinib in resistant cells and induced inhibition of cell growth in *in vivo* models of breast cancer. Consistent with this, CD36 expression was correlated with tumor grade. In addition, analysis of data from the NeoALTTO trial showed that CD36 increased in HER2-positive breast cancer after treatment with lapatinib and trastuzumab in the neoadjuvant setting and it was correlated with poor prognosis^[57]^ [Table 2].

Consistent with these findings, upregulation of CD36 expression induced resistance to gemcitabine in PDAC-resistant cells^[58]^ or chemoresistance in acute myeloid leukemia (AML) cells^[59]^ [Table 2].

Recently, promising results from a Phase I dose-escalation study demonstrated that CD36 is a predictive biomarker for treatment with VT1021 in patients with advanced solid tumors who are refractory to available therapies. VT1021 is a cyclic peptide that inhibits tumor growth by inducing the anti-angiogenic factor thrombospondin-1 (TSP-1) in myeloid-derived suppressor cells (MDSCs), thereby promoting TME modulation by simultaneously targeting CD36 and CD47^[60]^.

Taken together, these findings suggest that targeting CD36 may be a strategy to prevent tumor spread and metastasis. In addition, by promoting metabolic rewiring in cancer cells, CD36 may induce resistance to chemotherapy or targeted therapy, suggesting potential therapeutic strategies. FA uptake may also be an important marker of therapeutic response. Therefore, quantitative metabolic imaging of FA uptake in tumor cells may provide a potential additional diagnostic tool to improve patient stratification.

### Uptake of FAs affects immune response

If upregulation of CD36 levels on tumor cells is a prerequisite for malignant progression, this event on immune cells could dramatically impair the antitumor immune response. A recent study showed that CD36 levels were downregulated in cytotoxic CD8^+^ cells from melanoma patients responding to anti-PD1 treatment. Further studies have shown that CD36 upregulation in human CD8^+^ cells led to increased uptake of polyunsaturated fatty acids (PUFAs), rendering the cells more susceptible to ferroptosis-mediated cell death. Consistent with these findings, genes associated with lipid peroxidation or ferroptosis activation were shown to be enriched in tumor-infiltrating CD8^+^ cells with high CD36 levels compared to those with low CD36 expression. Interestingly, ferrostatin, an inhibitor of ferroptosis, increased the survival and antitumor activity of CD8^+^ cells *in vivo*. Moreover, the combination of CD36 inhibitors with anti-programmed cell death protein 1 (PD-1) antibodies resulted in successful antitumor therapy^[61]^ [Table 2], suggesting that CD36 is a critical target for improving the efficacy of immunotherapy.

Interestingly, it has been reported that one strategy used by tumor cells to evade immune control is to deprive cytotoxic T cells of the nutrients necessary to mount an effective antitumor response. Recently, it has been shown that cancer cells are able to take up significant amounts of cystine from the TME due to increased expression of Slc7a11, a component of the Xc transporter system, which plays a central role in glutathione homeostasis, a key player in antioxidant defense. This results in subsequent cystine deprivation in cytotoxic T cells and a dramatic impairment in glutathione synthesis, thereby increasing oxidative stress. In addition, cystine deprivation upregulated CD36, which in turn caused PUFA species to accumulate in the cell membrane of CD8^+^ cells, rendering them more susceptible to cell death by ferroptosis, consistent with the above data^[62]^. These data suggest that upregulation of CD36 can severely impair the functionality and the ability of CD8^+^ cells to mount a productive antitumor response.

Interestingly, elevated levels of CD36 have been identified in intra-tumor regulatory Treg cells in patients with melanoma, non-small cell lung cancer (NSCLC), and breast cancer. Subsequent studies have shown that CD36 supported immunosuppressive activities in regulatory T cells (Treg cells)^[63]^. Targeting CD36 on Tregs with specific inhibitors enhanced the efficacy of PD-1 therapy and stimulated antitumor immunity, consistent with the aforementioned data.

Human papillomavirus (HPV)-associated HNSCC is commonly treated with radiotherapy for localized, recurrent, and metastatic disease. A recent phase I/Ib clinical trial demonstrated that the combination of radiotherapy and anti-PD-L1 immunotherapy was effective in enhancing T-cell-mediated antitumor responses. Subsequent plasma metabolomic analyses showed that response to therapy may be correlated with increased FA metabolism and oxidative phosphorylation. In contrast, non-responders showed circulating levels of several free FAs and decreased levels of acyl-carnitines, indicating an impairment in FA oxidation, which is a major source of energy for cytotoxic CD8^+^ memory cells^[64]^. In light of these observations, a subsequent investigation in *in vivo* head and neck cancer (HNC) mouse models has shown that dietary or pharmacological modulation of FA catabolism modulated the antitumor response to radiotherapy. Treatment with fenofibrate (FF), an agonist of peroxisome proliferator-activated receptors α (PPARα) and a key regulator of FA metabolism, enhanced the efficacy of radiotherapy. Metabolomic analysis revealed that responders exhibited increased expression of enzymes involved in FA catabolism or beta-oxidation processes compared to non-responders. Conversely, the addition of high concentrations of oleic acid to the combination of FF and radiotherapy reduced treatment efficacy by promoting a Tregs-suppressing phenotype. Interestingly, upregulation of CD36 expression in Tregs cells was essential for maintaining suppressive functions^[65]^ [Table 2].

In conclusion, FA metabolism and oxidative phosphorylation are critical pathways in CD8^+^ cells. Further studies are needed to determine the translational potential of these findings and to elucidate the role of CD36 in different T cell subsets. Nevertheless, the results provide a theoretical basis for overcoming resistance to immunotherapy.

## OBESITY, TUMOR CELL AGGRESSIVENESS AND RESISTANCE TO CHEMOTHERAPY

Alterations in lipid metabolism in cancer cells are exacerbated in the context of obesity, where the accumulation of lipids and circulating free fatty acids (FFAs) occurs. Indeed, it is well established that individuals with a body mass index (BMI) above the normal range (overweight and obese) are at high risk of developing cancer. This is particularly relevant and significant for EC in women and rectal cancer in men^[66]^. Interestingly, an analysis of the immune profile of human EC showed a dramatic reduction in cytotoxic CD8^+^ cells that was associated with an increase in BMI, suggesting that metabolic dysregulation in the TME may, in principle, favor cancer progression^[67]^.

This is consistent with evidence in the literature that obesity may attenuate the efficacy of chemotherapy and immunotherapy, not only because of dose-limiting toxicity issues or increased risk of complications associated with the treatment^[68,69]^.

In addition to the clinical evidence, the molecular mechanisms that contribute to chemoresistance in the context of elevated lipids are not well defined.

**Figure 7:**
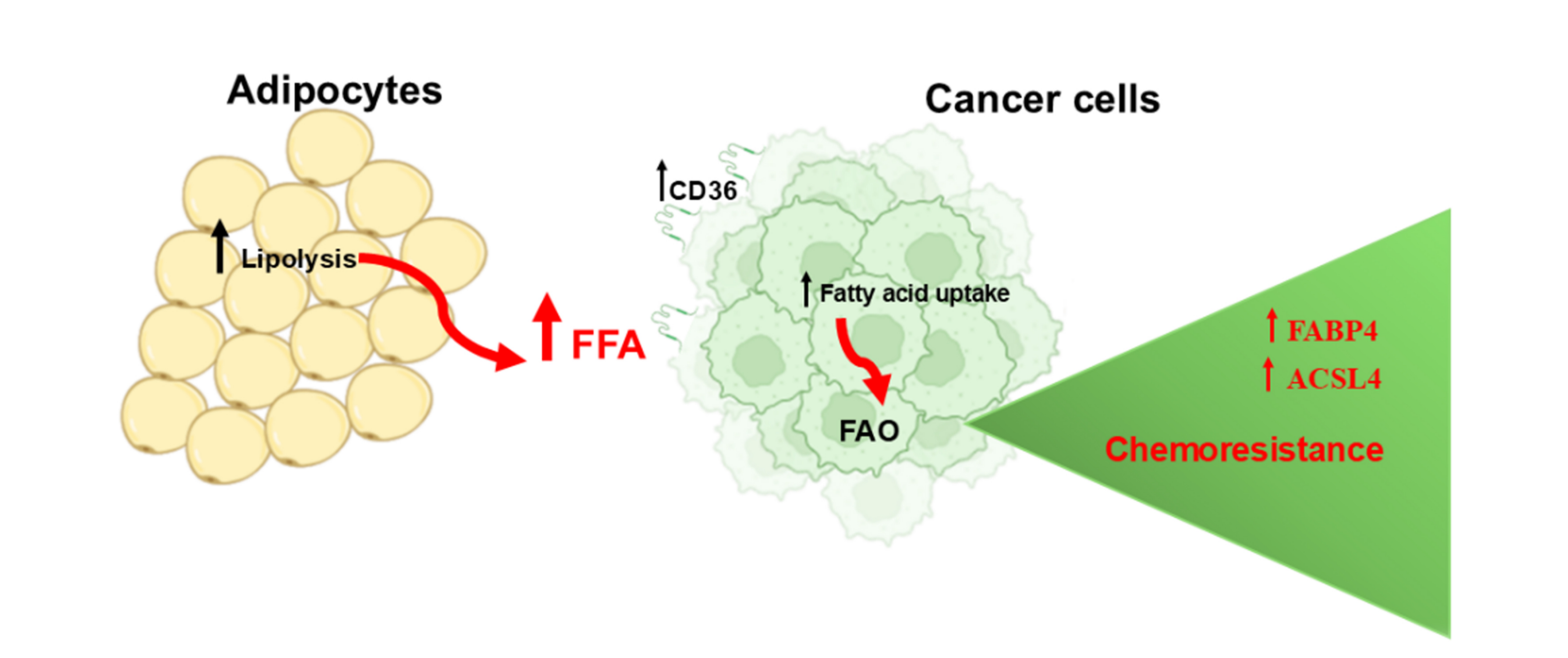
Uptake of FFAs promotes chemoresistance. Uptake of FFAs promotes FAO. Acetyl-CoA, a major product of FAO, increases phospholipid levels in mitochondria and improves their integrity. Upregulated FABP4 increases FAO and makes cancer cells more resistant to chemotherapy. Created with BioRender.com. FFA: Free fatty acids; FAO: β-fatty acid oxidation; FABP4: fatty acid-binding protein 4; ACSL4: long-chain acyl-CoA synthetase 4.

Several observations have suggested that the high availability of FFA in the obese adipose TME promotes aberrant FA metabolism in cancer cells by increasing intracellular FA metabolism (beta-oxidation) or storage as fuel, thereby meeting the energy demands for survival and malignant progression [Figure 7]. In addition, FA catabolism provides a protective mechanism against the lipotoxic effects associated with excessive intracellular FA uptake and mediated resistance to chemotherapy^[48]^. However, how FAO protects cancer cells from chemotherapeutic toxicity remains largely unclear.

A recent study by Li *et al.* showed that FAO promotes phospholipid synthesis in breast cancer cells by activating STAT3 in chemoresistant triple-negative breast cancer (TNBC)^[70]^. Specifically, acetylation of STAT3 by acetyl-CoA, one of the major products of FAO, promoted its transcriptional activity and upregulation of long-chain acyl-CoA synthetase 4 (ACSL4), which in turn increased phospholipid biogenesis. This led to an increase in phospholipids in mitochondrial membranes, which improved mitochondrial integrity and enabled cancer cells to survive chemotherapy-induced apoptosis [Table 3].

**Table 3:** Drug resistance metabolic biomarkers

**Biomarkers**	**Effect on cancer cells**	**Ref.**
ACSL4	↑ Chemoresistance	[70]
FABP4	↑ Chemoresistance	[71]
PIFAs	↑ Chemoresistance	[74]
AA	↑ Chemoresistance	[75,76]

ACSL4: long-chain acyl-CoA synthetase 4; PIFAs: Platinum-induced fatty acids; AA: arachidonic acid.

Moreover, it has been shown that OvCA cells, when cultured with primary human adipocytes, exhibit an altered lipidome profile and upregulation of lipid-binding proteins such as the lipid chaperone proteins FABP4 and CD36. Both proteins were found to be upregulated in peritoneal metastatic tumors compared to the primary tumor of patients with high-grade serous ovarian cancer (HGSOC).

Inhibition of FABP4 resulted in changes in the lipidomic profile of cancer cells. These changes included a reduction in FAO, an increase in lipid peroxidation and ROS generation, and a subsequent inhibition of cancer cell metastasis to the omentum. Notably, FABP4 mediated carboplatin resistance, while its inhibition increased the sensitivity of cancer cells to carboplatin, suggesting FABP4 as a therapeutic target for combination with platinum chemotherapy in patients with advanced or recurrent disease^[71]^ [Table 3].

In addition, genomic and transcriptomic profiles of obese versus lean subjects revealed molecular differences associated with a chronically inflamed TME and alterations in lipid metabolism such as arachidonic acid (AA) or cholesterol^[72]^. Consistent with these findings, increasing evidence supports the role of PUFAs in cancer risk and progression. Docosahexaenoic acid (DHA), eicosapentaenoic acid (EPA), and alpha-linolenic acid (ALA) are members of the n-3 family of PUFAs. DHA and EPA are the precursors of anti-inflammatory lipid mediators. Linolenic acid (LA) and AA are members of the n-6 family. AA is a known precursor of pro-inflammatory mediators^[73]^. A prominent work showing that PUFAs can mediate chemoresistance came from Emile Voest’s group in 2011^[74]^ [Table 3]. Mesenchymal cells were shown to systemically release platinum-induced fatty acids (PIFAs) from AA and EPA in a PLA2-, COX1-, and TXAS-dependent manner, which in turn increased the resistance of cancer cells to cisplatin and other chemotherapeutics (oxaliplatin, fluorouracil, and irinotecan).

A recent study has shown that AA may be a critical player in mediating the adaptive response of mesothelioma cells to chemotherapy. Antifolate treatment of mesothelioma cells induced cytosolic phospholipase A2 (cPLA2), which led to the release of AA from membrane phospholipids. AA activated NFKB pathways, which in turn induced a senescence-associated secretory phenotype (SASP) that correlated with chemoresistance in malignant pleural mesothelioma (MPM)^[75]^ [Table 3].

Furthermore, lipidomic analysis showed that adipocytes secreted AA, which induced chemoresistance to cisplatin in OvCA by activating the AKT pathway, suggesting that AA is an important mediator of chemoresistance in (OvCA). Notably, AA can be found in the circulation of cancer patients and may, therefore, be helpful in identifying patients who are prone to develop resistance to chemotherapy^[76]^ [Table 3].

This raises the intriguing possibility, still poorly explored to the best of our knowledge, that modulation of BMI, through dietary intervention or the use of drugs that target lipid metabolism, may influence response to conventional therapy (e.g., chemotherapy) and immunotherapy. Lipids act as early mediators of the adaptive response to therapy-induced stress, leading to the development of a pharmacologically resistant phenotype in tumors. Targeting specific mediators of resistance in cancer may contribute to progress toward more precise medicine and may be beneficial for obese cancer patients, who have significantly reduced survival. Identification of key mediators of resistance may also provide biomarkers as an adjunctive diagnostic tool. These studies may provide a promising prospective strategy to combat chemoresistance.

### Dynamic crosstalk between adipocytes and cancer cells

Adipocytes can also provide paracrine factors that reprogram the survival activities of cancer cells to adapt to external pressures, especially in the context of pharmacological treatment.

**Figure 8:**
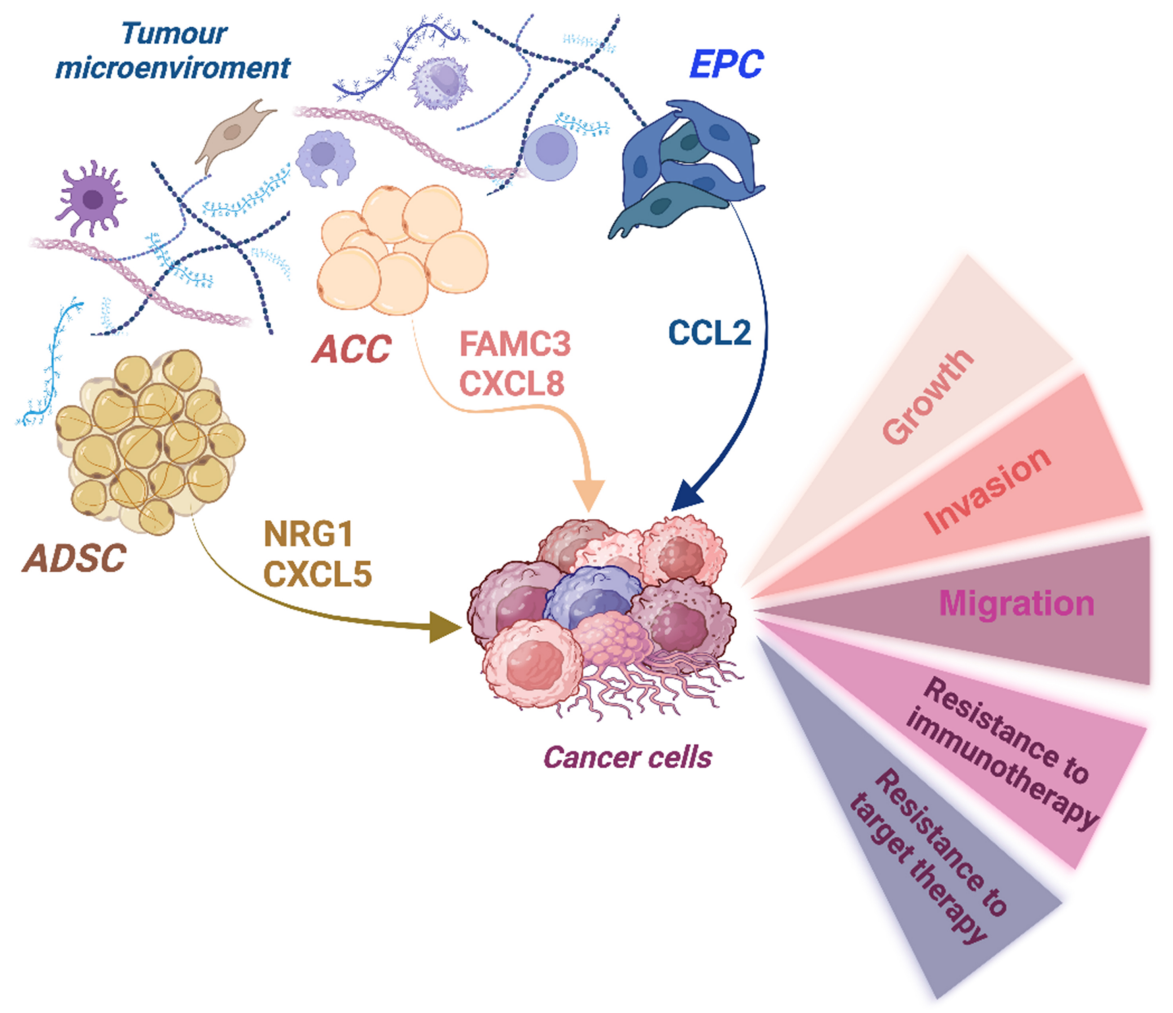
Paracrine signaling by TME on cancer cells. Cell subpopulations within the TME (ADSC, CAA, EPC) produce factors that support malignancy and drug resistance in cancer cells. Created with BioRender.com. TME: Tumor microenvironment; ADSC: adipose-derived stem cell; CAA: cancer-associated adipocytes; EPC: endothelial progenitor cell; NRG1: neuregulin 1; CXCL5: C-X-C motif chemokine ligand 5; FAM3C: metabolism-regulating signaling molecule C; CXCL8: CXC motif chemokine ligand 8; CCL2: CC motif chemokine ligand 2.

Recently, the FAM3 metabolism-regulating signaling molecule C (FAM3C) produced by cancer-associated adipocytes (CAA) was shown to promote a metastatic phenotype in breast cancer cells [Figure 8], consistent with data showing that upregulation of FAM3 was correlated with poor prognosis in several cancers^[77]^.

CAAs are fibroblast-like cells with reduced differentiation capacity and accumulation of small LDs^[78]^.

The CAAs may also be involved in the suppression of antitumor immunity through the secretion of factors that could profoundly remodel the TME. Adipocyte-secreted CXC motif chemokine ligand 8 (CXCL8, also known as IL-8) promoted a metastatic and immune evasive phenotype in BC cells through the activation of the PI3K/AKT pathway [Figure 8]. Targeting CAA-derived CXCL8 sensitized TNBC to anti-PD-1 immunotherapy^[79]^, which is consistent with data from the literature showing its role in inhibiting anti-PD-1 therapy in glioma^[79-81]^ [Table 4]. In addition, high serum levels of IL-8 have been associated with chemoresistance in gastric cancer patients^[82]^.

**Table 4:** Paracrine interactions between adipocytes and tumor cells

**Adipocyte-derived paracrine factors**	**Effect**	**Ref.**
FAM3C	↑ EMT and metastasis ↑ Serum FAM3C in metastatic BC Worse prognosis in BC patients	[77]
CXCL8	↑ EMT and metastasis ↑ Resistance to anti-PD-1 immunotherapy	[79]
NRG1	↑ Resistance to erdafitinib in bladder cancer	[83]
CXCL5	↑ EMT and metastasis Biomarker of advanced stage and metastasis in BC patients	[84]
POSTN	↑ Metastasis ↑ Chemoresistance	[86]

BC: Breast cancers; EMT: epithelial-to-mesenchymal transition; FAM3C: metabolism-regulating signaling molecule C; CXCL8: CXC motif chemokine ligand 8 neuregulin 1 (NRG1); CXCL5: C-X-C motif chemokine ligand 5; PD-1: anti-programmed cell death protein 1.

Adipocyte progenitor cells, also known as adipose-derived stem cells (ADSCs). ADSCs have been shown to produce and secrete neuregulin 1 (NRG1) in the TME. By activating human HER3, NRG1 induced resistance to erdafitinib in bladder cancer cells. Erdafitinib is a pan-FGFR tyrosine kinase inhibitor approved for metastatic urothelial cancer^[83]^.

Furthermore, resistin released from adipocytes in the TME stimulated ADSCs to release C-X-C motif chemokine ligand 5 (CXCL5) and promoted a metastatic mesenchymal phenotype in BC cells via activation of the ERK signaling pathway^[84]^. Notably, CXCL5 has been described as a predictive factor for resistance to sunitinib in metastatic renal cell carcinoma (mRCC) and lapatinib in HER2-positive breast cancer cells^[85]^.

Periostin (POSTN) produced by cancer cells induced secretion of the chemokine CC motif chemokine ligand 2 (CCL2) by endothelial progenitor cells (EPCs) [Figure 8], which in turn promoted a metastatic phenotype in HCC cells by increasing CD36 expression through the CCR2/STAT3 signaling pathway^[86]^ [Table 4]. Consistent with these data, POSTN has been shown to be induced by chemotherapy in TNBC and to support the survival and proliferation of mesenchymal chemoresistant cells^[87]^.

Altogether, this raises the possibility that the activation of both ADSCs and CAAs results in the creation of a pre-metastatic niche, a process that may be further enhanced in the context of obesity. This is particularly relevant when growth and metastasis occur predominantly near adipocytes, as observed in breast cancer (BC), or at anatomic sites where tumor cells are in close proximity to adipose tissue (AT), as is the case in OvCAs or colorectal cancers.

### Obesity and anti-cancer immunity

Moving to a more systemic ground, in the setting of a high-fat diet (HFD) and/or obesity, immune cells may rely on the same energy source as tumor cells, but cancer cells easily compete with immune cells. Therefore, the TME may be a competitive environment that promotes tumor cell proliferation and immunosuppression.

**Figure 9:**
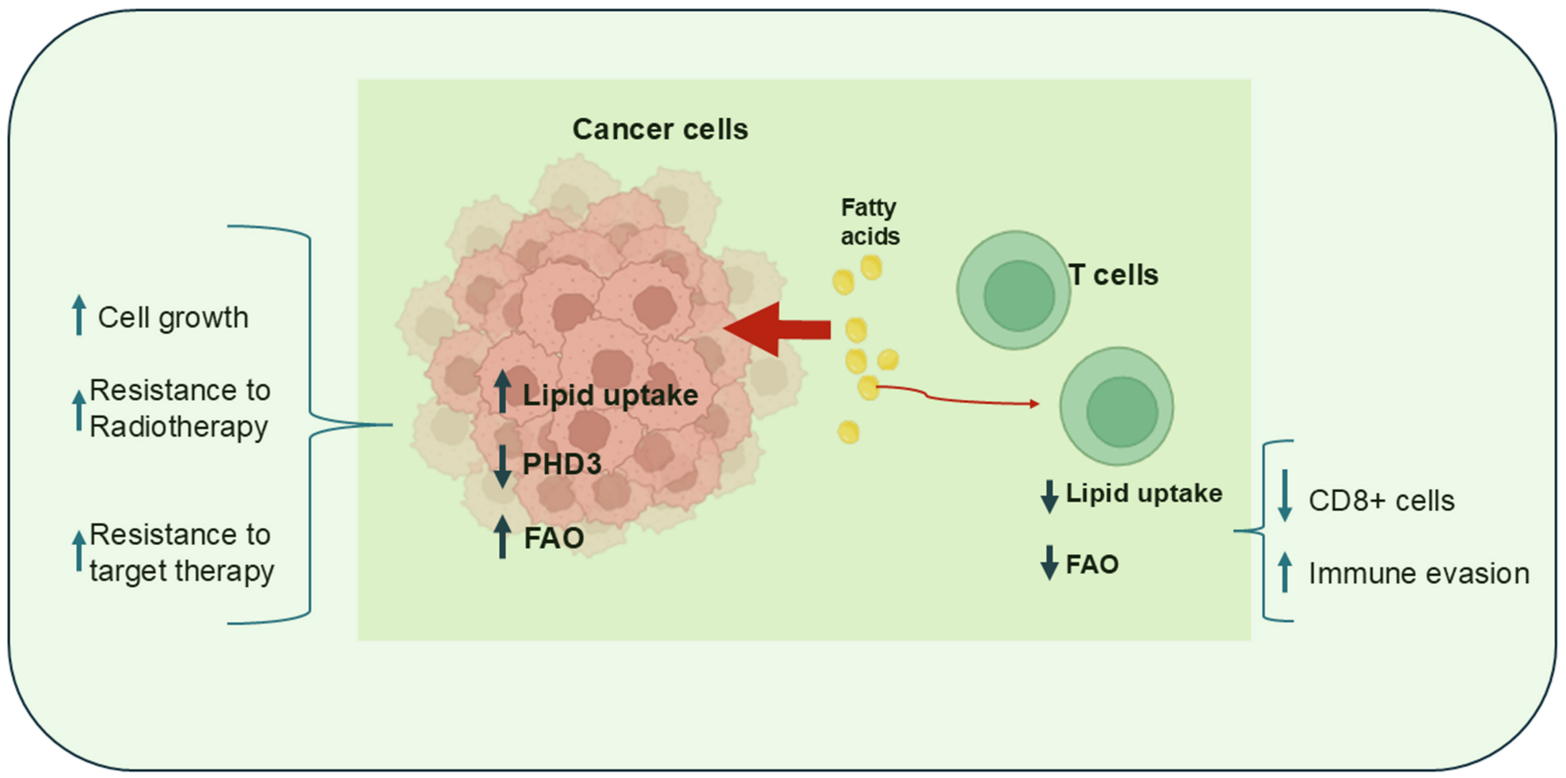
Competition for FA utilization between immune and tumor cells. Cancer cells compete with immune cells for FA uptake. FA uptake promotes FAO, which supports cell growth and resistance to treatment. PHD3 inhibits mitochondrial FA uptake. Created with BioRender.com. FA: fatty acid; FAO: β-fatty acid oxidation; PHD3: prolyl hydroxylase 3.

An elegant paper by Ringel *et al.* showed that obesity can alter metabolism in the TME by promoting tumor growth and inhibiting T cell function^[88]^. Specifically, using a syngeneic mouse model of colorectal adenocarcinoma, it was shown that obesity induced an increase in genes involved in FAO pathways and a concomitant decrease in the enzyme prolyl hydroxylase 3 (PHD3) in tumor cells. PHD3 is a key enzyme that inhibits mitochondrial uptake of free FAs. This event promoted cancer cell growth while inducing a reduction in the number and activity of cytotoxic CD8^+^ cells, which required lipids for energy production via the β-oxidation process [Figure 9]. Conversely, overexpression of PHD3 inhibited tumor growth and restored antitumor immunity, suggesting that CD8^+^ cells relied on the same energy source as tumor cells^[88]^.

Thus, obesity may induce systemic metabolic perturbations, resulting in severe immune cell dysfunction. Although these findings require further validation in human models, an interesting analysis of data from the Cancer Genome Atlas (TCGA) has shown low expression of PHD3 in obese patients with colorectal cancer (CRC) and, notably, in other human cancers, suggesting the intriguing possibility that blocking metabolic rewiring in tumor cells may improve antitumor immunity. In addition, PHB3 expression has been shown to enhance the efficacy of radiotherapy in pancreatic cancer cells^[89]^ and increase sensitivity to EGFR tyrosine kinase inhibitors in lung cancer cells [Figure 9], while its reduction has been shown to promote a mesenchymal phenotype in lung tumors^[90]^.

Diet-induced weight loss has been shown to be critical for restoring CD8^+^ cell function, as recently demonstrated in tumor-bearing obese mice. Notably, the antidiabetic drug semaglutide was not effective in rescuing antitumor immunity, highlighting the importance of both a healthy metabolic status and proper nutrition in restoring an efficient immune response. Importantly, tumors in these ex-obese animals were found to be responsive to ICB immunotherapy^[91]^. Thus, if immune dysfunction increases the risk of tumor development, it also leads to less efficient tumor editing, making tumors more immunogenic and susceptible to immune checkpoint blockade. Paradoxically, obesity may enhance the success of immunotherapy.

Taken together, these results suggest that metabolic challenges imposed by the TME mediate immune cell dysfunction and may drive tumor progression when nutrient competition between tumor and immune cells occurs due to limited access to nutrients. Therapeutic strategies that target the metabolic vulnerabilities of tumor cells may have beneficial effects in inhibiting disease progression and enhancing the antitumor immune response by combining lipid uptake with β-oxidation to promote CD8 T cell survival in the setting of lipid-rich tumors.

## CONCLUSIONS

During cancer progression, malignant cells undergo phenotypic and molecular changes induced by microenvironmental factors and/or pharmacological treatments, resulting in tumor heterogeneity and resistance to therapy. Metabolic reprogramming is a key feature of such a scenario, and both cancer cells and the TME play reciprocal roles in shaping metabolic adaptation to stress. This includes cancer progression toward an increasingly chemoresistant and immuno-evasive phenotype.

The TME could reprogram cancer metabolism by providing a plethora of essential nutrients and factors, which can lead to the formation of a specific milieu that supports the metabolic activities and survival of cancer cells throughout their development. Tumor cells, in turn, send various signals to the TME to create a pro-tumor and highly flexible microenvironment that is able to adapt to external pressures, especially in the context of pharmacological treatment.

Interestingly, the old idea of targeting the metabolic addictions of cancer cells also faces the possibility that host immune cells within the TME can be affected by metabolic intervention against cancer. The elucidation of these processes is, therefore, therapeutically meaningful, raising the importance of investigating a broad panel of cancer and TME targets and their potential for combinatorial therapy. This provides hope for future cancer trials and is one of the topics of the current work.

## DECLARATIONS

### Copyright

© The Author(s) 2024.
